# Using the Internet for Surveys and Health Research

**DOI:** 10.2196/jmir.4.2.e13

**Published:** 2002-11-22

**Authors:** Gunther Eysenbach, Jeremy Wyatt

**Keywords:** Clinical Trials, Confidentiality, Data Collection, Ethics, Research, Evaluation Studies, Informed Consent, Internet, Patient Selection, Qualitative Research, Research Design, Selection bias, Survey research, Research Subjects

## Abstract

This paper concerns the use of the Internet in the research process, from identifying research issues through qualitative research, through using the Web for surveys and clinical trials, to pre-publishing and publishing research results. Material published on the Internet may be a valuable resource for researchers desiring to understand people and the social and cultural contexts within which they live outside of experimental settings, with due emphasis on the interpretations, experiences, and views of `real world' people. Reviews of information posted by consumers on the Internet may help to identify health beliefs, common topics, motives, information, and emotional needs of patients, and point to areas where research is needed. The Internet can further be used for survey research. Internet-based surveys may be conducted by means of interactive interviews or by questionnaires designed for self-completion. Electronic one-to-one interviews can be conducted via e-mail or using chat rooms. Questionnaires can be administered by e-mail (e.g. using mailing lists), by posting to newsgroups, and on the Web using fill-in forms. In "open" web-based surveys, selection bias occurs due to the non-representative nature of the Internet population, and (more importantly) through self-selection of participants, i.e. the non-representative nature of respondents, also called the `volunteer effect'. A synopsis of important techniques and tips for implementing Web-based surveys is given. Ethical issues involved in any type of online research are discussed. Internet addresses for finding methods and protocols are provided. The Web is also being used to assist in the identification and conduction of clinical trials. For example, the web can be used by researchers doing a systematic review who are looking for unpublished trials. Finally, the web is used for two distinct types of electronic publication. Type 1 publication is unrefereed publication of protocols or work in progress (a `post-publication' peer review process may take place), whereas Type 2 publication is peer-reviewed and will ordinarily take place in online journals.

## Identifying issues for qualitative research

As the most comprehensive archive of written material representing our world and people's opinions, concerns, and desires (in industrialized countries), the Internet can be used to identify `issues' for qualitative (descriptive) research and to generate hypotheses. Material published on the Internet may be a valuable resource for researchers desiring to understand people and the social and cultural contexts within which they live--outside of experimental settings--with due emphasis on the interpretations, experiences, and views of `real world' people. Reviews of information posted by consumers on the Internet may help to identify health beliefs, common topics, motives, information, and emotional needs of patients, and point to areas where research is needed. Comparing recommendations found on the Web against evidence-based guidelines is one way to identify areas where there is a gap between opinion and evidence, or where there is a need for clinical innovation.

The accessibility of information for analysis and the anonymity of the Internet allow researchers to analyse text and narratives on Web sites, to use newsgroups as global focus groups, and to conduct interviews and surveys via e-mail, chat rooms, Web sites, or newsgroups.Topics suited to qualitative research include:

- Analysis of interactive communications (e.g. e-mail).
- Study of online communities (virtual self-help groups, newsgroups, mailing lists).
- Investigation of communication processes between patients and professionals.
- Study of consumer preferences, patient concerns, and information needs.
- Exploration of the `epidemiology of health information' on the Web [1-2].

The Internet population is unrepresentative of the general population, restricting the use of the Internet for quantitative studies (i.e. studies focusing on measurement). Qualitative studies, however, do not require representative samples:`In qualitative research we are not interested in an average view of a patient population, but want to gain an in-depth understanding of the experience of particular individuals or groups; we should therefore deliberately seek out individuals or groups who fit the bill' [3]. Three different research methodologies for qualitative research on the Internet may be distinguished:

- Passive analysis: For example, studying information on Web sites or interactions in newsgroups, mailing lists, and chat rooms--without researchers actively involving themselves.
- Active analysis: Also called participant observation; the researcher participates in the communication process, often without disclosing their identity as researcher. For example, they may ask questions in a patient discussion group implying that she or he is a fellow patient. Such studies often involve elements of deception, unless the researcher is a sufferer him- or herself.
- Interviews and surveys: See below.

Examples of these three types of qualitative research on the Internet are available elsewhere [1].

## Using the Internet for surveys

Using the Internet for surveys requires an awareness of methodologies, selection bias, and technical issues.

### Methodological issues

Internet-based surveys may be conducted by means of interactive interviews or by questionnaires designed for self-completion. Electronic one-to-one interviews can be conducted via e-mail or using chat rooms. Questionnaires can be administered by e-mail (e.g. using mailing lists), by posting to newsgroups, and on the Web using fill-in forms.

When e-mail is used to administer questionnaires, messages are usually sent to a selected group with a known number of participants, thus allowing calculation of the response rate. Surveys posted to newsgroups may request that the completed questionnaire is posted back to the researcher, but it is impossible to know who and how many people read the questionnaire. If Web-based forms are used, questionnaires can be placed in a password-protected area of a Web site (i.e. participation by invitation or registration only), or alternatively they may be open to the public (i.e. any site visitor can complete the survey). The latter option makes calculation of a response rate more difficult but not impossible: the number of people who access (without necessarily completing) the questionnaire is counted and used as the denominator. Web-based surveys have the advantage that the respondent can remain anonymous (as opposed to e-mail surveys, where the e-mail address of the responder is revealed). Furthermore, they are very convenient for the researcher, as responses can be directly stored in a database where they are immediately accessible for analysis.

Electronic interviews and surveys (`e-surveys') are emerging scientific research methodologies, pioneered by communication scientists, sociologists, and psychologists, although their use for health-related research is still in its infancy [4-10]. Examples of health-related research include:

- A Web-based survey on the effects of ulcerative colitis on quality of life [11].
- Collection of clinical data from atopy patients [12].
- A Web-based survey looking at complementary and alternative medicine use by patients with inflammatory bowel disease and Internet access [13].
- A survey of dentists regarding the usefulness of the Internet in supporting patient care [14,15].

**Guidelines for Web-based surveys**

**Scenarios that may be suitable for a Web-based survey**

**Respondent features:**

- Respondents are already avid Internet users; e-mail addresses known for reminder messages.
- Respondents are enthusiastic form fillers; will not require monetary incentives.
- Need for respondents covering a wide geographical area (e.g. rare clinical special ties, diseases).
- Respondents are known to match non-respondents and even non-Internet users on key variables.

**Survey features:**

- Need for complex branching, interactive questionnaire or multimedia as part of the survey instrument.
- Survey content will evolve fast (e.g. Delphi method surveys use repeating rounds of revised questionnaires delivered over a short period, incorporating aggregate results from previous rounds until convergence is achieved).
- Intent is to document bizarre, rare phenomena whose simple occurrence is of interest.
- No need for representative results: collecting ideas vs. hypothesis testing.

**Investigator features:**

- Limited budget for mailing and data processing, but good in-house Web skills.
- Precautions can be taken against multiple responses by same individual, password sharing.
- Web survey forms have been piloted with representative participants and demonstrate acceptable validity and reliability with most platform, browser, and Internet access provider combinations.
- Data is required fast in a readily analysed form.

**Scenarios that are unsuitable for a Web-based survey**

**Respondent features:**

- Target group is under-represented on Internet; e.g. the underprivileged, elderly people.
- Target group is concerned, however unreasonably, about privacy aspects.
- Target group requires substantial incentives to complete the survey.
- Need for a representative sample.

**Survey features:**

- Need for very accurate timing data on participants (inaccuracies in the range of seconds are added due to network transmission times, unless JavaScript or Java applets are used; see Glossary) or observational data on participants.
- An existing paper instrument has been carefully validated on target group.
- Need to capture qualitative data or observations about participants.
- Wish to reach the same group of participants in the same way months or years later.

**Investigator features:**

- Limited in-house Web or Java expertise but existing desktop publishing and mailing facility.

E-surveys may be part of a qualitative research process, but results can be analysed quantitatively as long as researchers are aware of potential bias (see below). In addition to gathering data, the Internet may also be used in the course of developing questionnaires, as it allows rapid prototyping and pilot testing of instruments, e.g. to evaluate the effect of framing the questions differently [16].

Several studies have checked the validity of Web-based surveys by comparing the results of studies conducted on the Web with identical studies in the real world. These seem to suggest that the validity and reliability of data obtained online are comparable to those obtained by classical methods [4,5,17-19]. However, issues of generalizability (mainly due to selection bias, discussed in detail below) remain important considerations, and the researcher should select his or her research question and interpret the results with care.The benefits and problems of Web-based surveys have been summarized by Wyatt, who suggests guidelines for when they may be appropriate (see Box 1) [20].

### Selection bias

In `open' surveys conducted via the Internet where Web users, newsgroup readers, or mailing list subscribers are invited to participate by completing a questionnaire, selection bias is a major factor limiting the generalizability (external validity) of results. Selection bias occurs due to:

- The non-representative nature of the Internet population.
- The self-selection of participants, i.e. the non-representative nature of respondents, also called the `volunteer effect' [21].

The non-representative nature of Internet demographics was briefly considered earlier. Considering whether the topic chosen for study is suitable for the Internet population is the first and probably the most important step in minimizing bias, thus maximizing response rates and increasing the external validity of the results [20]. For example, targeting elderly homeless alcoholics is unsuitable for an Internet survey and the results are likely to be heavily skewed by hoax responses.

Self-selection bias originates from the fact that people are more likely to respond to questionnaires if they see items which interest them, e.g. because they are affected by the items asked about, or because they are attracted by the incentives offered for participating. As people who respond almost certainly have different characteristics than those who do not, the results are likely to be biased.This kind of selection bias is more serious than the bias arising from the non-representative nature of the population, because the researcher deals with a myriad of unknown factors and has little opportunity to interpret his or her results accordingly. Such bias may be exacerbated via loaded incentives (e.g. typical `male' incentives such as computer equipment). Evidence suggests women are generally more interested in health topics and exhibit more active information-seeking behaviour [22], so are more likely to volunteer participation in health questionnaires. For Web surveys, the potential for self-selection bias can be estimated by measuring the response rate, expressed as the number of people completing the questionnaire divided by those who viewed it (cf. the participation rate, expressed as the number of site visitors viewing the questionnaire divided by the total number of site visitors).

### Technical issues

Although a detailed analysis is beyond the scope of this chapter, a synopsis of important techniques and tips for implementing Web-based surveys provides some insight into the difficulties faced by survey designers (see Box 2).

**Technical issues in implementing Web-based surveys**

**Use of `cookies'**

Cookies can assign a unique identifier to every questionnaire viewer, useful for determining response and participation rates, and for filtering out multiple responses by the same person. As cookies may be regarded with suspicion, we recommend that researchers openly state that cookies will be sent (and the reasons for this); set the cookie to expire on the day that data collection ceases; and publish a privacy policy.

**Measuring response time**

The time needed to complete a questionnaire can be readily calculated by subtracting the time a form was called up by the browser from the time it was submitted using an automatic time-stamp. The response time may be used to exclude respondents who fill in the questionnaire too quickly: this may identify hoax responses, where respondents don't read the questions.

**Avoiding missing data**

Forms can be configured to automatically reject incomplete questionnaires and point out missing or contradictory items. Checks can be made on the client (p. 9) prior to submission, or following submission to the server (where incomplete responses can also be analysed, e.g. during a questionnaire pilot).

**Maximizing response rate**

The number of contacts, personalized contacts, and contact with participants before the actual survey are the factors most associated with higher response rates in Web surveys [23]. Incentives increase the risk of selection bias (see text), but less so if cash is offered. Perhaps the best incentive (and the easiest to deliver via the Internet) is the promise of survey results or personalized answers (e.g. a score). The option to complete questionnaires anonymously avoids wariness associated with requests for personal information (e.g. an e-mail address), but increases the risk of hoax responses. Researchers should be open about who is behind the study, what the aim is, and provide opportunities for feedback. Although postal surveys are superior to e-mail surveys with regard to response rate, online surveys are much cheaper [24,25]. Schleyer [15] estimated that the cost of their Web-based survey was 38 percent less than that of an equivalent mail survey and presented a general formula for calculating break-even points between electronic and hard-copy surveys. Jones gave figures of 92 p per reply for postal surveys, 35 p for e-mail, and 41 p for the Web [24].

**Randomizing items**

Scripting languages may be used to build dynamic questionnaires (as opposed to static forms) that look different for certain user groups or which randomize certain aspects of the questionnaire (e.g. the order of the items). This can be useful to exclude possible systematic influences of the order of the items upon responses.

## Ethical issues

The ethical issues involved in any type of online research should not be forgotten [1,26-31]. These include informed consent as a basic ethical tenet of scientific research on human populations [32], protection of privacy, and avoiding psychological harm.

In qualitative research on the Web, informed consent is required when:

- Data are collected from research participants through any form of communication, interaction, or intervention.
- Behaviour of research participants occurs in a private context where an individual can reasonably expect that no observation or reporting is taking place, except when researchers do research `in public places or use publicly available information about individuals (e.g. naturalistic observations in public places, analysis of public records, or archival research)' [33].

The question therefore arises of whether researchers analysing newsgroup postings enter a `public place', or whether the space they invade is perceived as private. In the context of research, the expectation of the individual (whether he/she can reasonably expect that no observation is taking place) is crucial. Different Internet services have different levels of perceived privacy (in decreasing order of privacy: private e-mail; chat rooms; mailing lists; newsgroups;Web sites).The perceived level of privacy is a function of the number of participants, but also depends on other factors such as group norms established by the community to be studied. For example, in a controversial paper, Finn studied a virtual self-support group where the moderator was actively discouraging interested professionals who were not sexual abuse survivors from joining the group [34]. In those cases, obtaining informed consent (or seeking an ethical waiver, if the research could not practicably be carried out were informed consent to be required) is mandatory.

In practice, obtaining informed consent, especially for passive research methods, is difficult, as researchers usually cannot post an announcement to a mailing list or newsgroup saying that it will be monitored and analysed for the next few months, as this may greatly influence or even spoil the results, and because the mere posting of such a request may disrupt the community, and therefore be considered unethical. Researchers should therefore first obtain consent from a group moderator in order to explore whether even a request for permission is felt to be disruptive to the group process. If the moderator or person responsible for the list has no objections, one may then post a message to a newsgroup or mailing list explaining the purpose of the research, explaining that one will observe the community, assuring all participants of anonymity, and giving them the opportunity to withdraw from the newsgroup or mailing list or to exclude themselves from the study by writing to the researcher. The fundamental problem is that this may influence the communication process and may even destroy the community. Besides, participants who later join the group need to get the same information. An alternative would be to analyse the communication retrospectively and to write individual e-mails to all participants whose comments were to be analysed or quoted, asking for permission to use them; this technique has been used by Sharf [35].

In any case, researchers should make themselves familiar with the virtual community they are approaching; i.e. read the messages in a newsgroup for some time (`lurking'). Under no circumstances should researchers blindly spam (p. 31) or cross-post requests for research participation to various newsgroups.

Informed consent may also play a role when researchers report aggregate (collated and hence anonymous) data on usage patterns, such as a log-file analysis (reporting data on what Web sites have been accessed by a population). Crucial here is an appropriate privacy statement stating that these data may be analysed and reported in aggregate [28]. Note that aggregate data are exempt from the registration requirements of the UK's Data Protection Act of 1998.

In conducting surveys researchers may obtain informed consent by declaring the purpose of the study; disclosing which institutions are behind the study; explaining how privacy will be assured; and detailing with whom data will be shared and how it will be reported, before participants complete the questionnaire.

When reporting results, it is obvious that the total anonymity of research participants needs to be maintained. Researchers have to keep in mind that, by the very process of quoting the exact words of a newsgroup or mailing list participant, the confidentiality of the participant may already be broken as Internet search engines may be able to retrieve the original message, including the e-mail address of the sender. It is essential, therefore, to ask participants whether they agree to be quoted whenever there may be a retrievable archive, pointing out the risk that they may be identifiable. Problems can also potentially arise from just citing the name of the community (e.g. of a newsgroup), which may damage the community being studied.

## Finding methods, protocols, and instruments

For laboratory `bench work', researchers often need a protocol for a specific assay method. In addition to the possibility of searching literature databases, there are also specialized services on the Web that can assist in this research, such as MethodsFinder and the `Technical tips online' database at BioMedNet:

- **MethodsFinder (BIOSIS):** http://www.methodsfinder.org/
- **BioMedNet:** http://www.bmn.com/

Sometimes asking a specific question on the right newsgroup or mailing list is also very effective. Clinical researchers may be more interested in instruments to measure patient outcomes.An excellent guide to selecting quality-of-life instruments is the Quality of Life Instruments Database at the Mapi Research Institute: http://www.qolid.org/

Online statistical analysis tools are available at the Simple Interactive Statistical Analysis (SISA) Web site, while background information is available within the online book Statistics at square one:

- **SISA (Daan Uitenbroek):** http://home.clara.net/sisa/
- **Statistics at square one (British Medical Journal Publishing Group):** http://www.bmj.com/collections/statsbk/

Protocols of clinical trials, which may be useful for researchers developing their own protocols, can be found in some of the clinical trial databases available on the Web, as described below.

## Clinical trials and the Web

The Web is being used to assist in the identification and conduction of clinical trials.

### Identifying trials

To prevent unintended duplication of clinical research, detect underreporting of research, and ease the work of systemic reviewing, it has been suggested that we should prospectively register clinical trials [36-39]. It is, however, unlikely that there will ever be one complete centralized multinational database. Instead, multiple resources set up by numerous different organizations will exist [40]. Internet technology will play a central role in linking these databases and making this information available to researchers and patients. Some scenarios in which a search of trial databases may be useful:

- A researcher wants to conduct a randomized controlled trial and wants to know whether anyone else is already running one on the same topic.
- A physician has a patient who is asking about available trials.
- A patient is looking for ongoing trials.
- A researcher is looking for possible participants for his trial.
- A researcher doing a systematic review is looking for unpublished trials.

Information about ongoing and completed clinical trials is increasingly being published on the Internet, and searches on the Web may be a useful means of complementing traditional bibliographic searches if authors of systematic reviews wish to find ongoing or unpublished trials [41].

Researchers use their personal or department home pages to announce their interest in a certain research area or to recruit patients [42]. Journals like The Lancet have begun to publish research protocols on their Web site [43], and more and more researchers will also publish `pre-prints' (p. 239) of their findings on the Web [44].

Consumers and patient organizations also have an interest in disseminating information about ongoing trials; e.g. the National Alliance of Breast Cancer Organizations: http://www.nabco.org/

Government and funding agencies react to this need by establishing trial databases for consumers; e.g. the US National Institutes of Health searchable database [45]: http://ClinicalTrials.gov

Commercial enterprises also help researchers to recruit patients, or help patients to find clinical trials. For example:

- **CenterWatch Clinical Trials Listing Service (CenterWatch, Inc.):** http://www.centerwatch.com/
- **ClinicalTrialFinder.com (Clinical Data Technologies Ltd):** http://www.clinicaltrialfinder.com/
- **Current Controlled Trials (BioMed Central):** http://www.controlled-trials.com

Pharmaceutical companies and industry associations have likewise begun to recognize that openness and access to information on clinical trials and new drug developments can improve patient care and are part of social responsibility [46]. For example:

- **Clinical Trials Register (GlaxoSmithKline):** http://ctr.glaxowellcome.co.uk/
- **Search for Cures (Pharmaceutical Research and Manufacturers of America):** http://www.phrma.org/searchcures/

Finally, information or databases on ongoing clinical trials can often also be found on disease-specific sites. For example:

- **Canadian HIV Trials Network:** http://www.hivnet.ubc.ca/ctn.html
- **CancerNet (National Cancer Institute):** http://cancernet.nci.nih.gov/

### Conducting trials on the Web

The Web is increasingly being used in the course of conducting large-scale multi-centre clinical trials (e.g. for remote randomization and data entry), and in the distribution of information on trial progress or protocols [47,48]. Trial centres may enter patient data using Java applets (see Glossary) that encrypt data and send it to the data centre via the Internet [49-52], where the data are stored and randomized, returning for example a study number and randomization information.

## Pre-publishing and publishing research

Traditional publication is a well-defined event, whereas `publication' in the electronic age is much more of a continuum [53], reflecting and occurring during the entire research process from hypothesis formulation to data gathering, interpretation, and the presentation and discussion of the final results. In order to distinguish online collaborative `work in progress' from `final' peer-reviewed publication we may term the former `Type 1' and the latter `Type 2' electronic publication [54]. Here, peer review is not the distinguishing characteristic: in Type 1 publication a `post-publication' peer review process takes place. Type 2 publication will ordinarily take place in online journals. The following scenarios illustrate how researchers might use Type 1 electronic publication on the Internet:

- Sending and discussing preliminary results on mailing lists.
- Publishing drafts of scientific papers on pre-print/e-print sites (p. 239) in order to solicit comments and to improve the manuscript.
- Publishing data and information in databases; e.g. nucleotide sequences in the EMBL/Genbank databases.
- Publishing clinical trial protocols and raw data in a `trial bank' [55].

## Current awareness services

Electronic editions of paper journals and `stand alone' e-journals typically offer subscriptions to `TOC alerts', where users receive a table of contents by e-mail as soon as a new issue appears.The more sophisticated systems allow users to specify their interests using a controlled vocabulary, enabling the system to screen each newly published article for certain keywords or citations. Examples of current awareness services include:

- **Customised @lerts (British Medical Journal):** http://bmj.com/cgi/customalert/
- **JournAlert (Doctors.net.uk):** http://www.doctors.net.uk/
- **Journal Watch (Massachusetts Medical Society):** http://www.jwatch.org/
